# Beetle luciferases with naturally red- and blue-shifted emission

**DOI:** 10.26508/lsa.201800072

**Published:** 2018-08-16

**Authors:** César Carrasco-López, Juliana C Ferreira, Nathan M Lui, Stefan Schramm, Romain Berraud-Pache, Isabelle Navizet, Santosh Panjikar, Panče Naumov, Wael M Rabeh

**Affiliations:** 1 New York University Abu Dhabi, Abu Dhabi, United Arab Emirates; 2 Laboratoire Modélisation et Simulation Multi Echelle, MSME UMR 8208 CNRS, Université Paris-Est, Marne-la-Vallée, France; 3 Australian Synchrotron, Clayton, Australia; 4 Department of Biochemistry and Molecular Biology, Monash University, Melbourne, Australia

## Abstract

The different colors of light emitted by bioluminescent beetles that use an identical substrate and chemiexcitation reaction sequence to generate light remain a challenging and controversial mechanistic conundrum. The crystal structures of two beetle luciferases with red- and blue-shifted light relative to the green yellow light of the common firefly species provide direct insight into the molecular origin of the bioluminescence color. The structure of a blue-shifted green-emitting luciferase from the firefly *Amydetes vivianii* is monomeric with a structural fold similar to the previously reported firefly luciferases. The only known naturally red-emitting luciferase from the glow-worm *Phrixothrix hirtus* exists as tetramers and octamers. Structural and computational analyses reveal varying aperture between the two domains enclosing the active site. Mutagenesis analysis identified two conserved loops that contribute to the color of the emitted light. These results are expected to advance comparative computational studies into the conformational landscape of the luciferase reaction sequence.

## Abstract (precis)

New crystal structures of red- and green blue–shifted beetle luciferases reveal that the color emission mechanism is dependent on the active site microenvironment affected by the conformation of loop regions.

## Introduction

The dazzling flashes of bioluminescent light communicated by fireflies have inspired scientists, writers, artists, and laymen for centuries. Beyond its visual appeal, bioluminescence is an irreplaceable bioanalytical tool for in vivo imaging, monitoring of cell proliferation, studies into protein folding and secretion, environmental research, and food quality control. At the core of this natural phenomenon lies a fundamental process of energy transduction by luciferase enzymes that convert the chemical energy stored within the ground-state substrate (luciferin) to an excited, emissive state of the product (oxyluciferin) by a spin-forbidden process (Johnson & Shimomura, 1972; DeLuca, 1976). Although beetle luciferase systems share identical substrates and chemical reaction sequence, they emit a range of different colors from yellow green (λ_max_ ≈ 560 nm), which is typical for common firefly species, such as the North American firefly *Photinus pyralis* (G_Pp_) and the Japanese firefly *Luciola cruciata* (G_Lc_), to orange and even red (λ_max_ = 590–623 nm) from certain click beetles and railroad worms (Viviani et al, 1999, 2011; Ugarova & Brovko, 2002). The molecular origin of the different colors of light emitted by different luciferases remains the most elusive mechanistic aspect of this photochemistry and has been continuously debated since the 1970s. In the absence of structural information on WT luciferases that emit light other than green, several mechanisms have been advanced and subsequently refuted. Current mechanistic models that are based on experimental and computational analysis on available green-emitting luciferase structures remain inconclusive (Hosseinkhani, 2011).

Here, we describe the first crystal structures of two rare WT luciferases from Brazilian beetles that emit light with exceptional colors; a luciferase from the head lanterns of the glow-worm *P. hirtus* (Coleoptera: Phengodidae), the only known luciferase that naturally emits red light (λ_max_ = 623 nm; RE_Ph_), and a green-emitting luciferase from the firefly *A. vivianii* (Coleoptera: Lampyridae) that displays a blue-shifted emission relative to common firefly luciferases (λ_max_ = 538 nm at pH 8; GB_Av_) (Viviani et al, 1999, 2008, 2011). Biochemical and structural analyses of the two luciferases, combined with computational modeling, provide the best insight yet into the relationship between the structure and color of light emitted by beetle luciferases.

## Results

### Structural determination of the red-emitting luciferase from *P. hirtus*

**Figure S1.**
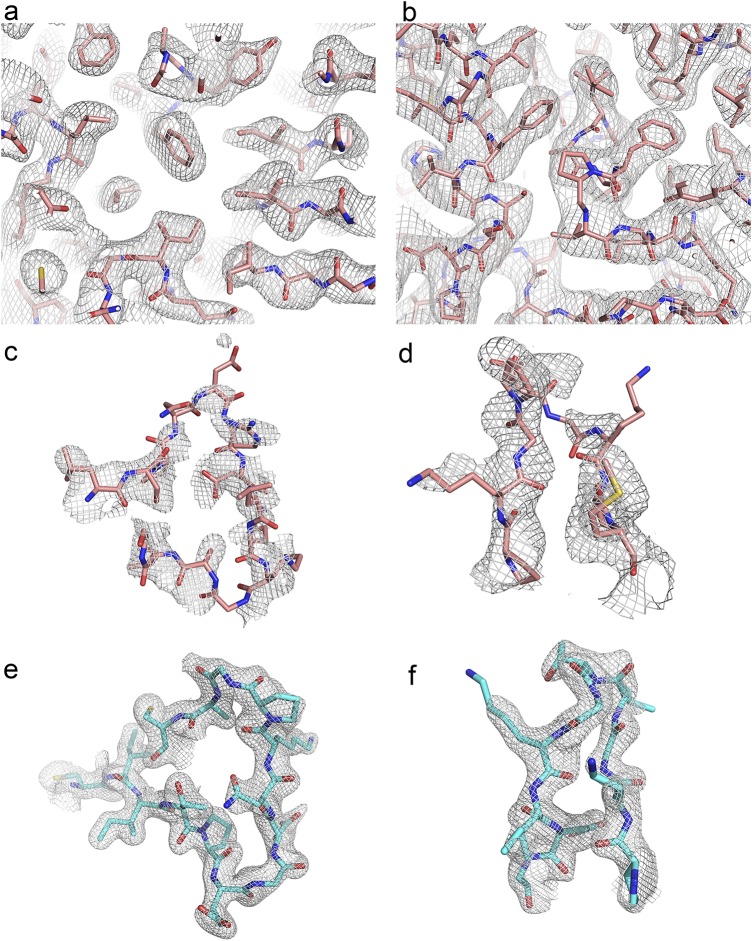
2FoFc electron density maps contoured at 1 sigma and the corresponding atomic models. **(A)** RE_Ph_ *P*1 crystal form at 3.05 Å resolution showing a random region of the protein and its electron density map around the core. **(B)** RE_Ph_ *P*3_1_21 crystal form solved at 3.6 Å resolution showing random region of its atomic model and electron density map around the core of the protein. **(C)** Loop^348–361^ in RE_Ph_ fitted within the electron density. Only molecules C and F of the *P*1 crystal had sufficient quality in the electron density to trace the complete loop. **(D)** Loop^521–528^ in RE_Ph_ fitted within the electron density map of the *P*3_1_21 crystal form. Only molecule B of the *P*3_1_21 crystal form has enough quality to trace the C-terminal domain that was not traceable in any of the molecules in the *P*1 crystal form. **(E)** Loop^351–364^ in GB_Av_ fitted within the electron density. **(F)** Loop^523–530^ in GB_Av_ fitted within the electron density.

**Figure 1.**
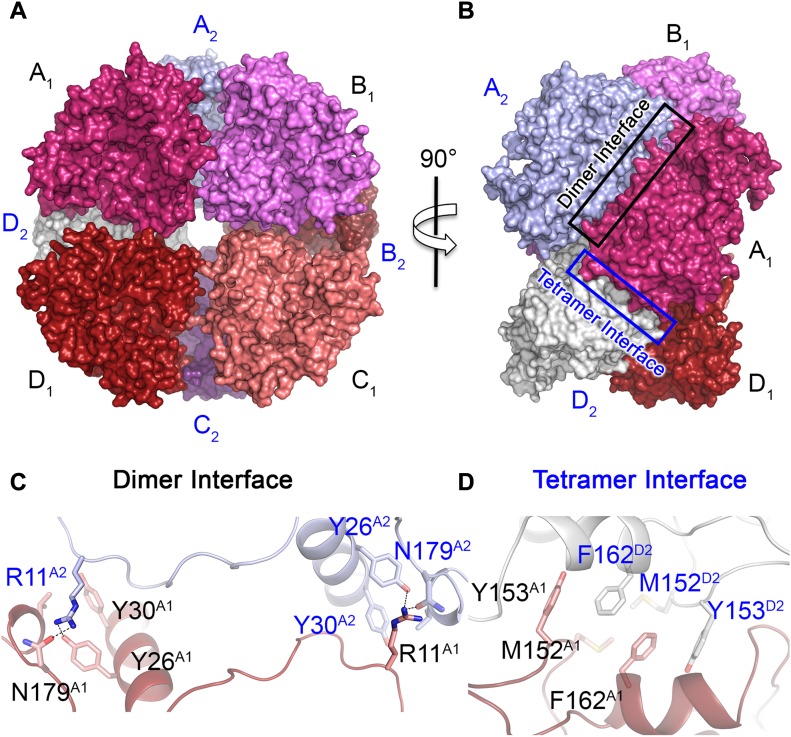
Crystal structure of the naturally red-emitting luciferase from *P. hirtus* (RE_Ph_). **(A)** Front view of the partial octameric conformation found in the *P*1 crystal form (only the N-terminal domains were observed in the electron density maps). **(B)** Lateral view of the octamer that highlights the surface interactions between the monomers. **(C)** Close-up view and interactions across the dimer interface between monomers A1 (red) and A2 (blue). The interacting residues are shown as stick models with matching colors as monomers A1 and A2 in panels (A, B). The two surfaces are related to each other by a *C*_2_ axis. The broken lines show the interactions between residues R11, N179, and Y26. Mutation R11A disrupted the octamer to give monomers in solution. **(D)** Close-up view of the tetramer interface across the dimers, which are assembled as an octamer (monomer A1 is dark red and monomer D2 is white). The interactions between the two surfaces are predominantly hydrophobic interactions between Y153, M152, and F162 from both dimers. Similar to the dimer interface, the surfaces at the tetramer interface are related by a *C*_2_ axis. Single and double mutations at residues Y153 and F162 generated exclusively dimers in solution.

**Figure S2.**
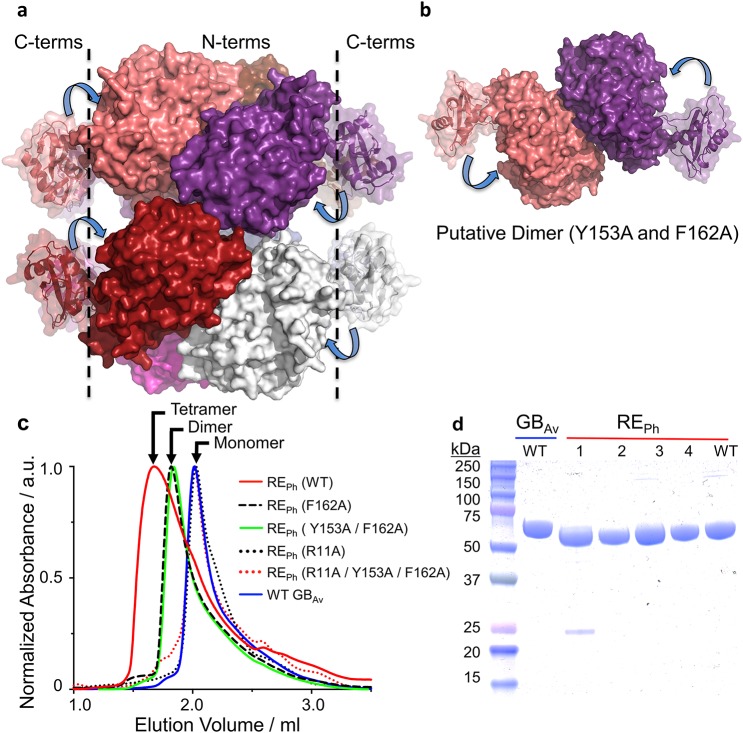
Oligomerization of red-emitting luciferase RE_Ph_. **(A)** The octamer of RE_Ph_ shows the N-terminal domains packed to form the core of the octamer and the C-terminal domains pointing outward. The dotted lines separate the N- from C-terminal domains in the complex and the arrows indicate the direction for the movement of the C-terminal domains during catalysis. The flexibility of the C-terminal domain is important for opening and closing of the active site. **(B)** Putative dimer of RE_Ph_ in its double mutant Y153A/F162A as part of the dimer interface. **(C)** Size-exclusion chromatogram of WT RE_Ph_ shows a peak with an estimated MW of ≈240 kD that corresponds to the tetramer. The single mutants F162A or Y153A in the tetramer interface of RE_Ph_ produced only dimers with an estimated MW of ≈122 kD. The combination of R11A in the dimer interface with Y153A/F162A in the tetramer interface produced monomers as a single peak at 61 kD, which is identical to WT GB_Av_. However, the R11A alone was sufficient to disrupt the octameric assembly of the WT RE_Ph_ and produced exclusively monomeric peak similar to the WT GB_Av_ luciferase. All MWs were estimated by using MW gel filtration calibration kit on Superdex 200 5/150 GL (GE Healthcare). **(D)** SDS–PAGE analysis of the GB_Av_ WT and the RE_Ph_ WT and mutant protein samples after completing the protein purification. The RE_Ph_ mutants are as follows: lane 1: R11A; lane 2: R11A, Y153A, and F162A; lane 3: Y153A and F162A; and lane 4: F162A. Coomassie stain was used to visualize the protein bands and ImagJ was used to quantity the protein samples' purity >90%.

The crystal structure of WT RE_Ph_ was determined at low resolution by molecular replacement from two different crystal forms in the space groups *P*1 and *P*3_1_21 at resolution of 3.05 Å and 3.60 Å, respectively (Table S1). Both crystal forms presented good-quality electron density maps, which were improved by the non-crystallographic symmetry of both unit cells (Fig S1A and B). Unlike previously reported luciferases that are exclusively monomeric (Conti et al, 1996; Franks et al, 1998; Nakatsu et al, 2006; Auld et al, 2010; Cruz et al, 2011; Sundlov et al, 2012; Kheirabadi et al, 2013; Branchini et al, 2017), in the *P*3_1_21 crystal form, RE_Ph_ exists as tetramer, although in the *P*1 crystal form it is an octamer in the asymmetric unit (Figs 1A and B, S2A, and B). The N-terminal domains in the octamer core structure are assembled as a tetramer of dimers and packed over dimer and tetramer interfaces, and the C-terminal domains point outward. This assembly accounts for the structural flexibility and increased thermal motion of the C-terminal domains which is apparent from the residual electron density. The inability to model the C-terminal domain of firefly luciferases in certain crystal conditions, as result of its high flexibility, has been previously shown (Auld et al, 2010; Thorne et al, 2012; Kheirabadi et al, 2013). Only one out of the four C-terminal domains was observed in the density maps of the *P*3_1_21 crystal form and none of the eight C-terminal domains of the RE_Ph_ octamer could be resolved in the *P*1 crystal form (Supplementary Note 1). The RE_Ph_ structure is consistent with the α/β-fold of other beetle luciferases and the substrate-binding pocket in each of the monomers is located between the larger (N-terminal) and smaller (C-terminal) domains. A displacement of the C-terminal domain can open and close the active site in a conformational rearrangement that is purportedly triggered by binding of the substrates (Nakatsu et al, 2006).

Table S1 Data collection and refinement statistics.

Supplementary Note 1 In the structure of RE_Ph_, we were able to build the C-terminal domain of only one of the four molecules that were observed in the asymmetric unit of the *P*3_1_21 crystal form. The C-terminal domain showed higher average *B* factors (69.4 Å^2^, with several regions with values above 100) relative to the N-terminal domain within the same crystal form (60.3 Å^2^). The electron density maps of the C-terminal domains in some of the molecules were incomplete, and in other cases they showed very little to no definite electron density. The redundancy imposed by the non-crystallographic symmetry aided in the building and refinement of a reliable model, particularly of the N-terminal domain, and improved the agreement between the structural model and the determined electron density (Supplementary Fig. 1a and 1b).

In the structure of RE_Ph_, each dimer within the octamer is stabilized by multiple hydrogen bonds between amino acid residue R11 from one of the monomers and Y26, Y30, and N179 from the other monomers, which extend over a *C*_2_ axis across the dimer interface (Fig 1C). The interactions across the dimer interface are strong electrostatic interactions with two contact points contributed by R11 from each monomer (Fig 1C). With the low resolution of the RE_Ph_ structure, site-directed mutagenesis clearly confirms these interface interactions, where the single mutation (R11A) was sufficient to disrupt the octamer of RE_Ph_ and resulted exclusively monomers in solution (Fig S2C and D). On the other hand, the hydrophobic interactions between M152, Y153, and F162 from two dimers contribute to the weak overall interactions between the dimers over the tetramer interface (Fig 1D). As confirmed by mutagenesis and size-exclusion chromatography, the interactions across the tetramer interface are weaker relative to the interaction of the individual monomers over the dimer interface. The WT RE_Ph_ exists as a tetramer in solution (Supplementary Note 2). Consequently, mutations on the tetramer interface, which include single (F162A) and double (Y153A and F162A) mutants, produced only dimers in solution (Fig S2C). Overall, the emission of WT RE_Ph_ remains unaffected by interface mutations that result in fragmentation of the RE_Ph_ octamer into dimers and monomers; we therefore conclude that the red emission of RE_Ph_ is not a result of its quaternary structure. Instead, the red light is intrinsic to the structural fold of the RE_Ph_ monomer and originates from the specific packing and microenvironment of its active site.

Supplementary Note 2 The crystal structure of RE_Ph_ is an octamer in the *P*1 crystal form and a tetramer in the *P*3_1_21 crystal form (Fig. 1a and 1b; Supplementary Fig. 2a and 2b). The octamer of RE_Ph_ is composed of dimers that are joined at the tetramer interface (Fig. 1c and 1d; Supplementary Fig. 2b). The tetramer is formed by interactions between M152, Y153 and F162 from the dimers (Fig. 1d). The degree of oligomerization of RE_Ph_ in solution was confirmed by using size-exclusion chromatography (SEC; Supplementary Fig. 2c and 2d). The estimated molecular weight (MW) of the wild-type (WT) RE_Ph_ from the SEC analysis was ≈ 240 kDa, which corresponds to the tetramer found in the *P*3_1_21 crystal. The octamer with a predicted MW of 480 kDa was not observed in solution, which is probably a result of weak inter-tetramer interactions that dissociate in solution, but maybe stabilized at higher protein concentrations represented in the crystallization drops.
Mutations at the tetramer interface, which include double mutant Y153A and F162A, produced dimers with MW of 122 kDa. The monomer was not detected for any of the mutations at the tetramer interface. However, the monomer units within the dimers are held strong polar interactions at two contact points on the dimer interface, where R11 from one of the monomers forms H-bonding interactions with Y26, Y30, and N179 of the other monomer (Fig. 1c). The single mutant R11A in the dimer interface was sufficient to disrupt all interface interactions and exclusively generated monomers of RE_Ph_ with a MW of 61 kDa as determined by SEC analysis (Supplementary Fig. 2c). Interestingly, disrupting the dimer interface was sufficient to disassemble the tetramer interface. These results indicate that the overall interactions across the dimer interface are relatively stronger than those across the tetramer interface, and that the dimer is more stable than the tetramer. This can also explain why the octamer was not observed in solution as it might be less stable than the tetramer with more hydrophobic interface interactions.

### Structural determination of the blue-shifted green emission luciferase from *A. vivianii*

**Figure 2.**
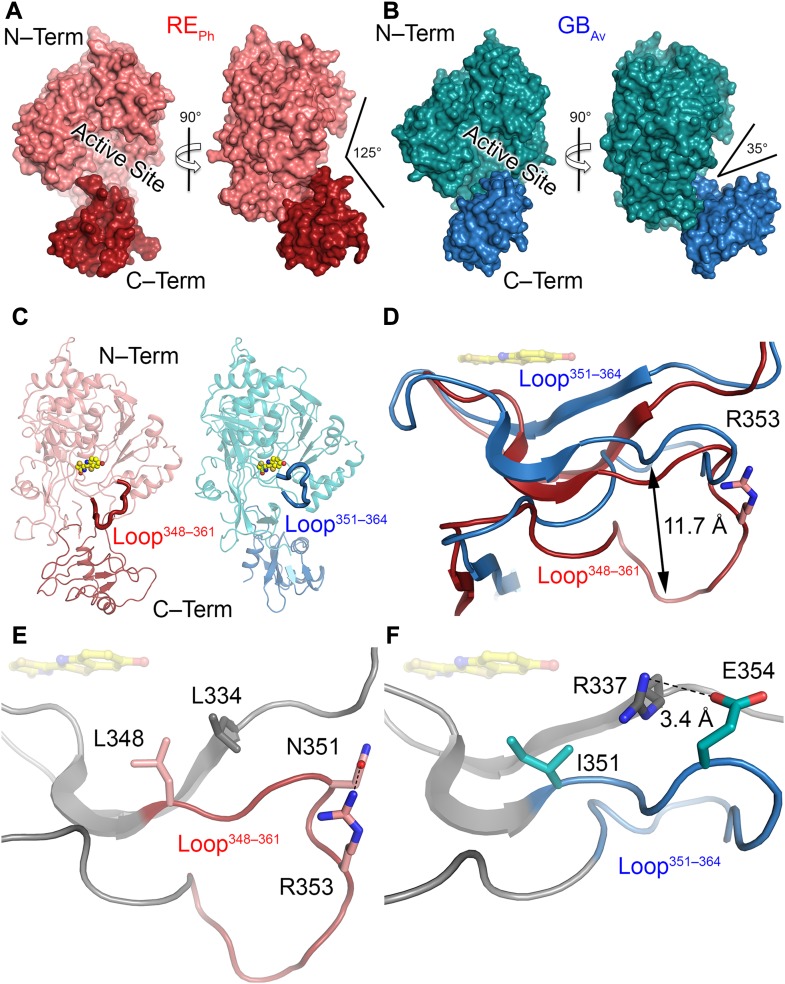
Analysis of the structures of red-emitting RE_Ph_ and blue-shifted green-emitting GB_Av_ luciferases. **(A)** Full-length structure of molecule B, the only monomer with a complete C-terminal domain of RE_Ph_ in the *P*3_1_21 crystal form (the structures of the other three monomers in this crystal lack the C-terminal domain, which could not be observed in the difference electron density maps). The conformation of RE_Ph_ has the largest aperture between the N-terminal (“N-Term”) and C-terminal (“C-Term”) domains among the luciferases with known crystal structures. **(B)** Structure of one of the two monomers in the asymmetric unit of GB_Av_ (the monomer of GB_Av_ with a larger aperture is shown here; the angle of the aperture of the other monomer is 30°). The structural packing of both GB_Av_ monomers is less open relative to RE_Ph._ The RMSD value of the superimposed monomers is 0.22 Å based on all α-carbons in the structure (the deviations were prominent in the C-terminal domain, with RMSD of 2.1 Å). Identical conformations were found for the N-terminal domain, with RMSD based on the α-carbons of 0.08 Å. **(C)** The loose packing of loop^348–361^ (red) in the N-terminal domain of RE_Ph_ relative to the tight packing of loop^351–364^ (blue) in the N-terminal domain of GB_Av_ (see Fig S6). **(D)** The shift of loop^348–361^ (red) in RE_Ph_ relative to loop^351–364^ (blue) in GB_Av_ by superposition of the two monomers based on the α-carbons of the whole structures (Fig S6A). R353 (pink sticks) is the only known insertion in the RE_Ph_ sequence. **(E)** Natural substituted residues found in loop^348–361^ (pink) of RE_Ph_ close to the active sites are shown in gray and pink sticks. **(F)** The relatively conserved residue counterparts in loop^351–364^ (blue) of GB_Av_ are shown in gray and blue sticks. In panels D, E, and F, the reaction product (oxyluciferin, shown with yellow sticks) is shown by superimposing the structures reported here with the structure of G_Lc_ in complex with oxyluciferin (PDB code: 2D1R).

**Figure S3.**
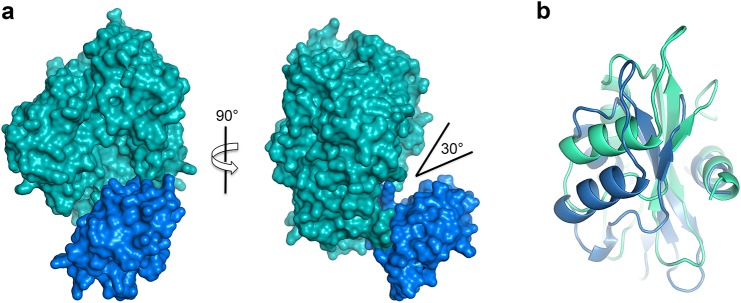
Two molecules in the asymmetric unit (AU) of the GB_Av_ crystal. **(A)** General structure of molecule “A” in the AU of the GB_Av_ crystal represented by its molecular surface. The C-terminal domain (highlighted in blue) and its N-terminal domain (cyan) have a different conformation relative to the other molecule “B” in the AU. In this case, the angle that describes the space between the domains is lower and, therefore, the overall conformation is less open for molecule “A” than molecule B. **(B)** View of the C-terminal domains of both molecules when superimposed. The different conformations of the C-terminal domains are clear when the two molecules (B in blue cartoons and A in cyan) are superimposed, with a rotation along the center of the main β-sheet.

**Figure S4.**
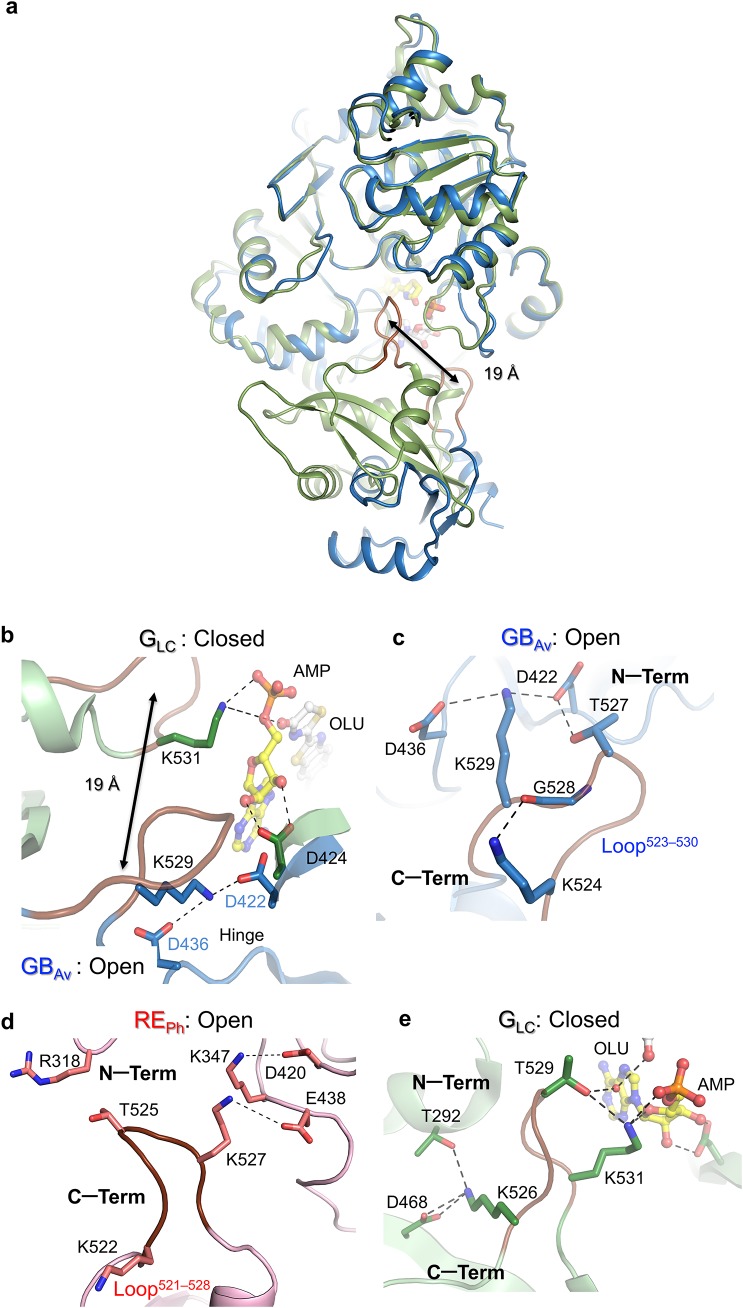
Analysis of the conformation of loop^523–530^ GB_Av_ and loop^521–528^ RE_Ph_ in “open” conformations against loop^525–532^ G_Lc_ in complex with bioluminescence product oxyluciferin (OLU) and AMP (PDB code:2D1R), which represents the “closed” conformation of luciferase. **(A)** Superimposition of GB_Av_ (blue ribbons) and G_Lc_ (green ribbons). Loop^523–530^ (highlighted in brown in both structures) is displaced ≈19 Å upon closure of the active site. **(B)** A close-up view that shows the displacement of loop^523–530^ in G_Lc_ relative to GB_Av_. In the open conformation (GB_Av_), loop^523–530^ interacts with the hinge (D436, blue sticks) and D422 of the active site through K529 (blue sticks). Upon binding of the substrate, a large conformational rearrangement closes the active site to produce a conformation similar to that of G_Lc_ in complex with the products. In the closed conformation, K531 and D424 (green sticks) of G_Lc_ interact with OLU and AMP, and these interactions may be the driving force for the conformational change that closes the active site. **(C)** Interactions of loop^523–530^ GB_Av_ in the open conformation with the hinge loop connecting the N- with the C-terminal domain. K529 of the loop interacts with D436 and D422 of the hinge loop and the N-terminal domain, respectively. T527 of the loop interacts with D422 in the N-terminal domain. The structure of the loop is maintained by interactions between K524 and the backbone carbonyl oxygen of G528. **(D)** Loop^521–528^ RE_Ph_ in the C-terminal domain of RE_Ph_ is more open and more distant from the N-terminal domain relative to GB_Av_. Loop^521–528^ RE_Ph_ is less efficiently packed and lacks many of the interactions with the N-terminal domain (see Fig S6A). However, the interaction between K527 and E438 is maintained, and it is similar to K529 and D436 of GB_Av_. **(E)** In the closed conformation, loop^525–532^ of G_Lc_ is shifted closer to the substrate-binding site for direct interaction between K531 of loop^525–532^ with the α-phosphate of the nucleotide. Moreover, T529 of loop^525–532^ interacts with OLU through a water molecule. Loop^525–532^ of G_Lc_ also stabilized the closed conformation through interactions with T292 and D468 of the N- and C-terminal domains, respectively.

To identify structural features that are important in the color tuning mechanism, the crystal structure of GB_Av_ luciferase with a blue-shifted green emission of *l*_max_ = 538 nm at pH 8 was determined for comparison with the RE_Ph_ (Fig 2B and C). The emission of GB_Av_ is at higher energy relative to the well-studied luciferases G_Lc_ (Nakatsu et al, 2006) and G_Pp_ (Conti et al, 1996) that emit green-yellow light. The GB_Av_ crystals diffract to a resolution of 1.9 Å when free of substrate and crystallize in a space group *P*2_1_2_1_2_1_ with two independent GB_Av_ monomers in the asymmetric unit (Fig S1E and F and Table S1). Although the N-terminal domains of the two GB_Av_ molecules are very similar (root-mean-square deviation [RMSD] value of 0.1 Å calculated on all backbone atoms), their C-terminal domains have different conformations with an RMSD value of 2.1 Å (Fig S3A and B). Thus, although the luciferase in both molecules can be considered as being in its “open” conformation, the luciferase with the smaller aperture is maybe an intermediate between the “open” and “closed” conformations (Figs 2B, S3A, and B), the latter being attributed to the structure of G_Lc_ in complex with the reaction products (Nakatsu et al, 2006). Indeed, a superposition of the N-terminal domains of the “open” conformations of GB_Av_ and RE_Ph_ with the green-emitting luciferase G_Lc_ in complex with oxyluciferin and AMP (Protein Data Bank [PDB] code: 2D1R), which is in its “closed” state (Nakatsu et al, 2006), indicates a movement of the C-terminal domain of about 10 Å to 30 Å between the two states to open/close the active site (Fig S4). The pronounced flexibility of the C-terminal domain of GB_Av_ was further examined by classical molecular dynamics simulations in which the C-terminal domain required 5 to 15 ns to shuffle between the two conformations. Together with the structural data, these results confirm the pronounced mobility of the C-terminal domain of beetle luciferases, which is capable of reversible opening and closing of the active site through two catalytic conformations during the bioluminescence reaction. The two catalytic conformations are stimulated by rotation on the C-terminal domain of firefly luciferases (Sundlov et al, 2012). Notably, the structures of both GB_Av_ and RE_Ph_ have wider openings than G_Pp_ luciferase devoid of substrates or products (PDB codes: 1LCI and 5DV9; Figs 2A and B, and S4A) (Conti et al, 1996; Wu et al, 2017). The aperture of the active site with an angle of ∼125° between axes crossing the center mass of each domain (*P*3_1_21 crystal form) shows that RE_Ph_ is the most open conformation among the known luciferase structures, which corresponds to the high mobility of its C-terminal domain. This feature indicates that the flexibility of the C-terminal domain may play a role in tuning the color of light emitted.

### Loop^351–364^ is important for the green and red emission of GB_Av_ and RE_Ph_

**Figure S5.**
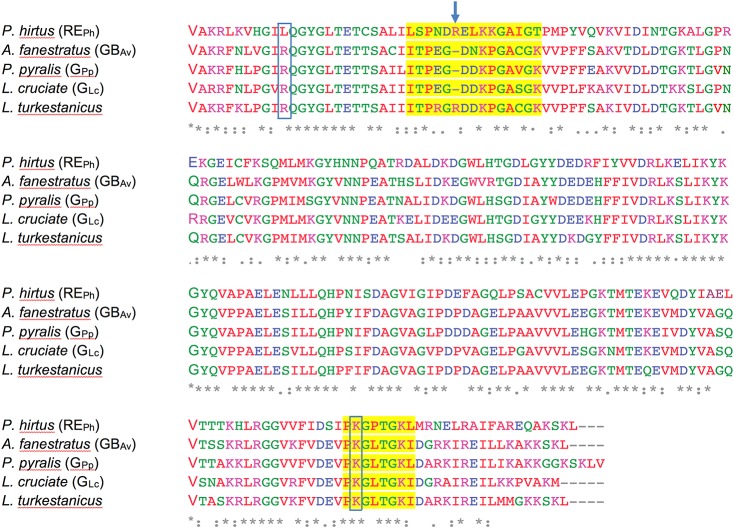
Amino acid sequence alignment of beetle luciferases. The amino acid sequence alignment of loop^348–361^ and loop^521–528^ in RE_Ph_ and loop^351–364^ and loop^523–530^ in GB_Av_ was performed against luciferases from *P. pyralis* (G_Pp_), *Luciola cruciate* (G_Lc_), and *Lampyris turkestanicus* using Clustal Omega. The loop regions are highlighted in yellow. Amino acids R337 and K524 that altered the emission color of GB_Av_ are boxed. Amino acid R353 insertion in RE_Ph_ is indicated with an arrow that is also found in *L. turkestanicus* luciferase.

**Figure S6.**
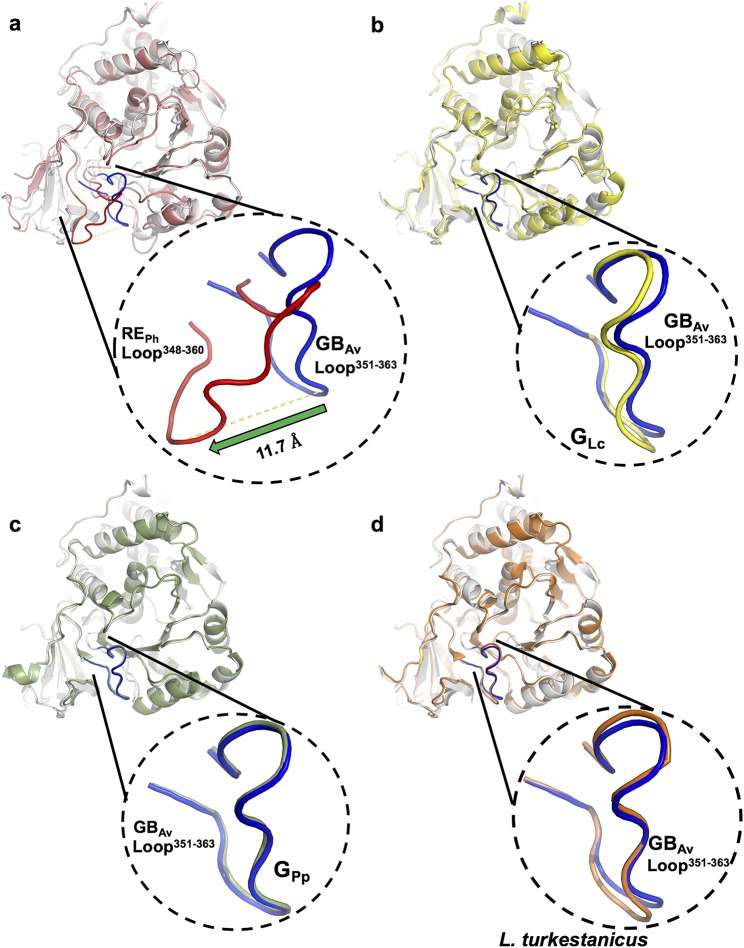
Structural superimposition of loop^351–364^ in GB_Av_ with the corresponding loop in RE_Ph_ and other beetle luciferases. Superimposition in ribbons representation of GB_Av_ (white) against (A) RE_Ph_ (red); (B) G_Lc_ (yellow; PDB code: 2D1R); (C) G_Pp_ (green; PDB code: 3IES); (D) Luciferase from *Lampyris turkestanicus* (orange; PDB code: 4M46). The loop^348–361^ RE_Ph_ (red) is displaced by ∼12 Å in comparison with the same loop^351–364^ GB_Av_ (blue). On the other hand, loop^351–364^ GB_Av_ has same conformation as the corresponding loops in green-emitting luciferases G_Lc_, G_Pp_, and luciferase from *L. turkestanicus* (panels B–D).

**Figure S7.**
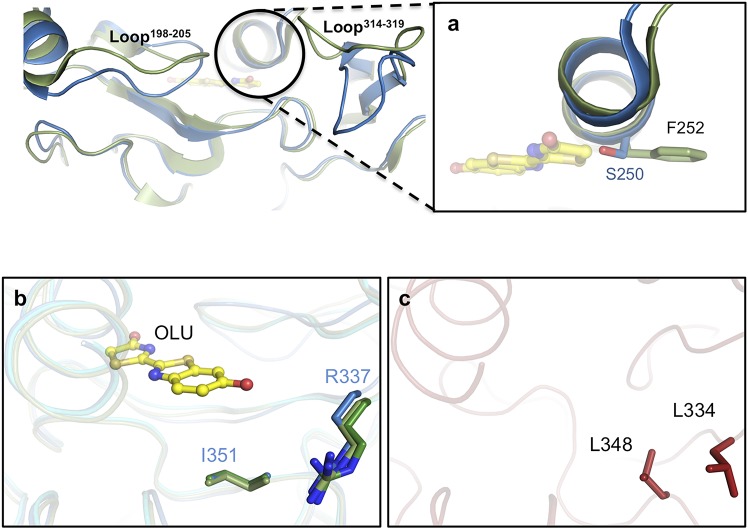
Analysis of the active sites of GB_Av_ (blue ribbons) and G_Lc_ (green ribbons). Superposition of these structures indicates that two loops (loop^198–205^ and loop^314–319^) in the blue-shifted green-emitting luciferase (GB_Av_) show a conformational change relative to the structure of green-emitting G_Lc_ in its closed conformation. **(A)** A close-up view of the helix above the active site shows the lateral chains (sticks) of residues in different conformations in the two structures. GB_Av_ has a single substitution, S250, in the active site instead of F252 in G_Lc_. **(B)** Residues I351 and R337 (in sticks color-coded according to the structure) are conserved in G_Lc_ and have identical conformations in the three determined crystal structures. The main structures are superimposed and shown in dark green (G_Pp_), pale green (G_Lc_), and blue ribbons (GB_Av_). Oxyluciferin in complex with G_Lc_ is shown as a ball-and-stick model. **(C)** Residues L334 and L348 from RE_Ph_ (red sticks) are not conserved in the different green-emitting luciferases that are substituted by arginine and isoleucine, respectively.

**Figure 3.**
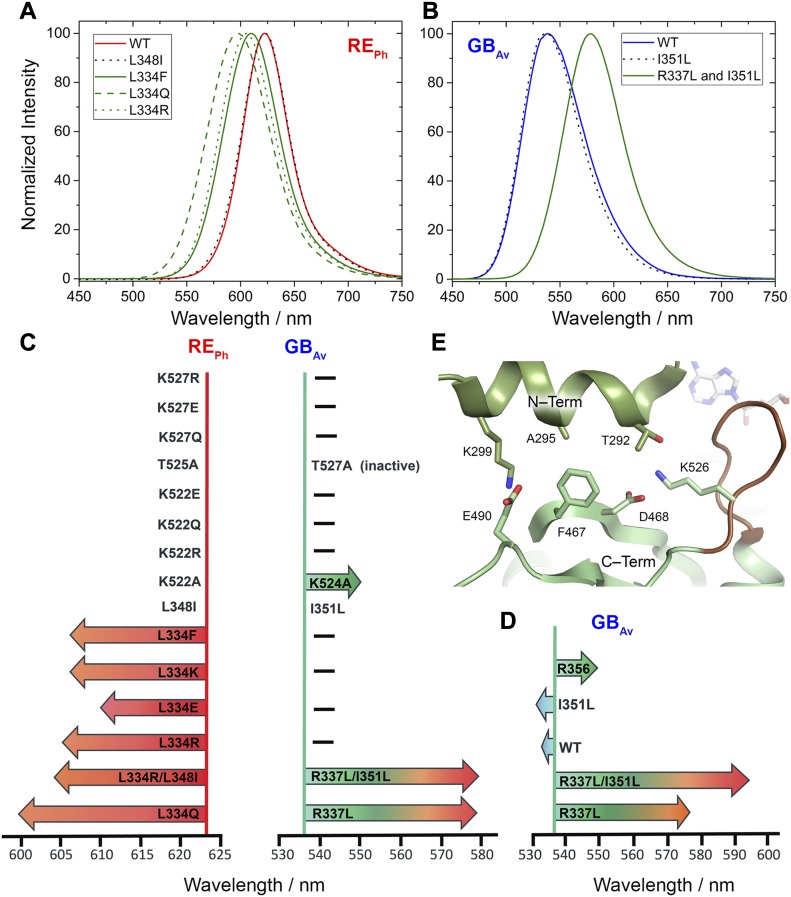
Normalized bioluminescence emission spectra and kinetics of WT and mutants of luciferases RE_Ph_ and GB_Av_. **(A)** At pH 8.0, the emission from WT RE_Ph_ with λ_max_ = 623 nm was blue-shifted between 600 and 610 nm in mutants of L334. **(B)** At pH 8.0, GB_Av_ emission at λ_max_ = 538 nm for the WT enzyme was red-shifted to 580 nm in double mutant R337L/I351L. Single mutant I351L did not alter the emission of GB_Av_. **(C, D)** Schematic of the experimental (C) and calculated (D) data for mutation-induced shifts of emissions of GB_Av_ and RE_Ph_ (Tables S2, S3, S4, and S5). The vertical *y*-axis is set at the WT emission of GB_Av_ or RE_Ph_. Each arrow represents a mutant (labeled inside the arrow) that shifts the color from the WT emission and points in the direction of change of the emitted color. The tip of the arrow is a qualitative representation of the color shift and the maximum emission wavelength. The mutations that did not affect the color emitted by the WT luciferase are labeled immediately next to the *y*-axis. **(E)** Residues at the interface (green sticks) between the N- and C-terminal domains in the closed conformation of G_Lc_. Previous mutations on residues at this interface (E490 and F467) and mutant K524A in GB_Av_ (K526 in G_Lc_) reported here red-shifted the color between 10 and 15 nm.

Detailed analyses of the structures of RE_Ph_, GB_Av_, and G_Lc_ revealed multiple amino-acid residues that may be directly involved in determining the color of emitted light (Supplementary Note 3). The most remarkable structural feature is the conserved loop^351–364^ in GB_Av_ (which corresponds to loop^348–361^ in RE_Ph_) that is located in the N-terminal domain at the edge of the active site (Figs 2C and D, and S5). In addition, the presence of the only known insertion in beetle luciferases (R353 in RE_Ph_; Fig S5), the proximity of loop^351–364^ to the active site and several key substitutions around the benzothiazole-binding region, which were previously found to have an impact in the color tuning (Viviani et al, 2007), drove us to investigate the relevance of loop^351–364^ in the color-tuning mechanism. The loop^348–361^ in RE_Ph_, albeit at low resolution, was modeled in the electron-density map (Fig S1C). In GB_Av_, loop^351–364^ is tightly held by strong ionic interactions between E354 on the loop and R337 on the N-terminal domain (Figs 2F, S6, and S7). Residue K358 on the opposite side of the loop also interacts with D427 and D429 from the terminal section of the N-terminal domain (for figure clarity, these interactions are not shown in Fig 2F). The ionic interactions on both sides of loop^351–364^ fix its position relative to the enzyme backbone. This stability is reflected in the low RMSD value for the loop backbone atoms of only 0.3 Å between GB_Av_ and G_Lc_. These interactions are absent in RE_Ph_ owing to the replacement of R337 and E354 in GB_Av_ with L334 and N351 in RE_Ph_, respectively (Figs 2E, S5, and S6A). The absence of these strong interactions with the enzyme core (represented by R337) increases the mobility of analogous loop^348–361^ in RE_Ph_, as reflected in the higher RMSD value of 2.3 Å for the respective atoms between the RE_Ph_ monomers. Notably, RE_Ph_ is also the only beetle luciferase with an additional residue, R353, in loop^348–361^ (Figs 2E and S5). Although insertion of R353 in green-emitting luciferases including, GB_Av_ (Fig 3D and Table S2), red-shifts the color that is emitted (Tafreshi et al, 2007; Alipour et al, 2009), its deletion from the red-emitting RE_Ph_ as demonstrated here and previously does not affect the red emission (Viviani et al, 2007).

Table S2 TD-DFT/MM emission for electronic transition between S_1_ and S_0_ for the model GB_Av_-closed-insert-R356 (resulting from the insertion of Arg 356). TD-DFT/MM done with 6-311G(2d,p) basis set and B3LYP functional on structure optimized at the same level of theory.

Supplementary Note 3 The origin of the difference in color of emitted light was investigated by introducing mutations in RE_Ph_ and GB_Av_ at the following positions:(1) Loop^348‒361^ in RE_Ph_, which corresponds to loop^351‒364^ in GB_Av_(2) Loop^521‒528^ in RE_Ph_, which corresponds to loop^523‒530^ in GB_Av_(3) Single mutants in the C-terminal domain, which includes S462F, E486K, and E486V in RE_Ph_, and F465S, E488K, and E488V in GB_Av_, which were found previously to alter the color emitted by luciferase from *Luciola mingrelica* (Modestova et al, 2014; Modestova & Ugarova, 2016).
Multiple mutants of the C-terminal domain introduced here did not alter the emission of both RE_Ph_ and GB_Av_ luciferases. However, loop^348‒361^ was found to play an important role in determining the color of emitted light of RE_Ph_ and GB_Av_. This loop is tightly packed against the N-terminal domain of GB_Av_ and other green-emitting luciferases relative to RE_Ph_ (Fig. 2c; Supplementary Fig. 6). Its high mobility in RE_Ph_ is a consequence of the restructuring of the hydrogen bonding as a result of the natural substitutions and insertions (Fig. 2c).
After detailed structural and sequence analysis of the luciferases, only few natural substitutions were found within the active site. Introduction of I351L, one of the natural substitutions found, did not change the emission of WT GB_Av_ (Fig. 3b). However, introduction of R337L red-shifted the emission of WT GB_Av_ from 538 to 580 nm (Fig. 3c), and it drastically decreased its intensity. Furthermore, the intensity of R337L was slightly enhanced after the introduction of a second mutation, I351L, which did not alter the emission of WT GB_Av_ on its own. Similar to the single mutant (R337L), the double mutant (R337L/I351L) shifted the emission from 538 to 580 nm. Therefore, R337 is clearly important the red-emission, and the substitution I351L enhances the intensity without affecting the emission energy.
Various mutations were introduced at position L334 in RE_Ph_ to investigate its role in color emission. All mutants at L334 blue-shifted the emission of RE_Ph_ from 623 nm for the WT enzyme to 610‒606 nm for L334R, L334K, L334E, L334Q, and L334F (Fig. 2e and 3a). The single mutant with the strongest blue shift (from 623 to 600 nm) is L334Q. The contribution of a second amino acid L348 does not appear to be significant in the fine-tuning of the color, because introduction of L348I mutation in RE_Ph_ did not alter the WT emission energy (Fig. 3a and 3c).
The role of loop^523‒530^ in the C-terminal domain was investigated by characterizing the role of K524, T527, and K529 in the stability and color emission of GB_Av_ and its corresponding positions in RE_Ph_, K522, T525, and K527, respectively (Supplementary Fig. 4). Loop^523‒530^ is important for the opening and closing of the active site, and could play a role in the color of the emitted light and the enzyme stability. None of the mutants introduced in loop^521‒528^ of RE_Ph_, which includes K522R, K522E, K522Q, T525A, K527R, K527E, K527Q, and K527A, have altered the WT emission (Fig. 3C). Although K524A red-shifted the emission of GB_Av_, T527A and K529A did not alter its emission (Fig. 3C; Supplementary Fig. 7b). Furthermore, residue K524 plays an important role in the stabilization of the closed conformation. In the closed conformation of G_LC_ in complex with oxyluciferin and AMP (PDB code: 2D1R) (Nakatsu et al, 2006), K526 in the C-terminal domain interacts with T292 in the N-terminal domain. The tight H-bonding interactions between K526 and T292 contribute to the microenvironment and tight packing around the active site of G_LC_ as is the case for GB_Av_ (Supplementary Fig. 4e). These interactions are absent in RE_Ph_ (Supplementary Fig. 4d) because T292 of GB_Av_ and G_LC_ is substituted with P287 in RE_Ph_.
Thermal unfolding analysis by differential scanning calorimetry (DSC) measurements revealed different thermodynamic stabilities with calculated melting points (*T*_m_) of 35°C for WT RE_Ph_ and 43 °C for WT GB_Av_. Although similar thermodynamic stability for the GB_Av_ enzyme was determined in the absence or presence of substrates (*T*_m_ ≈ 43°C; Supplementary Fig. 7e and 7h), the addition of substrates changed the shape of thermograms and increased the stability of the WT RE_Ph_ from 35°C to 41°C with luciferin (Supplementary Fig. 7c and 7g). Two transitions were observed for the melt of RE_Ph_ in the presence of ATP with T_m_ values of 36°C and 41°C.
The introduction of mutant K524A in GB_Av_ slightly decreased its thermal stability relative to the WT enzyme with similar overall shapes of the thermograms (Supplementary Fig. 7e and 7f). However, the introduction of mutant K522A changed the overall shape of the thermogram of WT RE_Ph_ (Supplementary Fig. 7c and 7d). In addition, the thermal melt of K522A RE_Ph_ was not detectable in the absence of substrates and the signal was detectable only after the addition of substrate, luciferin or ATP.
The emission of WT GB_Av_ was pH-dependent, with a blue shift from 550 nm at pH 7.0 to 538 nm at pH 8.0 (Supplementary Fig. 7b). The introduction of K524A in GB_Av_ red-shifted the WT emission by ≈ 18 nm, from 550 to 573 nm at pH 7.0 and from 538 to 555 nm at pH 8 (Supplementary Fig. 7b). Increasing the pH above 8.0 did not alter the emission of GB_Av_, but a decrease in pH below 7.0 inactivated the enzyme. The emission of RE_Ph_ was pH-independent for both WT and the K522A mutant with *λ*_max_ = 623 nm (Supplementary Fig. 7a).

Additional biochemical and computational analyses were performed to assess the effects of R337 on the emission color because our and others’ (Viviani et al, 2007; Viviani et al, 2016) mutation experiments indicated that it has an important role in determining the emitted color (Fig 3C and D and Supplementary Notes 3 and 4 and Tables S3, S4, and S5). Mutation of R337L in green-emitting GB_Av_ red-shifted its emission by 42 nm, from 538 to 580 nm (Fig 3B). The theoretical calculations reproduced the observed trend and estimated a red shift of ≈60 nm, from 535 to 577 nm (Table S4, snapshot 4). A second mutation, I351L, did not affect the emission energy of R337L, which shows the dominating effect of R337 (Table S3). These results confirm that the interactions of R337 with loop^351–364^ in GB_Av_ are critical for the emission of green light. We anticipate that this effect is more general for green-emitting luciferases and an analogous mutation that disrupts these interactions would shift their green emission (λ_max_ ≈ 560 nm) and thereby decrease the emitted energy ≈ 40–60 nm to generate emission of red light (≈600–620 nm). Thus, it appears that the absence of strong interactions of L334 in red-emitting luciferase RE_Ph_ (analogous to R337 in GB_Av_) is critically important for its red emission. In support of this hypothesis, replacement of L334 (mutation L334R) in red-emitting RE_Ph_ blue-shifted the light of this luciferase by 18 nm, from 623 to 605 nm without a significant change in light intensity (Fig 3A). Similarly, the double mutant L334R/L348I of RE_Ph_ blue-shifted emissions from 623 to 600 nm, although the single mutation L348I did not affect the emission maximum, which confirms that L334 in RE_Ph_ is the key residue that determines the low energy of its emission. Nevertheless, these mutants still emit red light. We hypothesize that synergistic effects that involve residues outside the active site could stabilize the closed state and shift the emission further to the green.

Table S3 TD-DFT/MM emission for electronic transition between S_1_ and S_0_ for the model GB_Av_-closed-I351L (resulting from the I351L mutation in GB_Av_-closed before MD). The TD-DFT/MM calculations were performed with the 6-311G(2d,p) basis set with the B3LYP functional on a structure optimized at the same level of theory.

Table S4 TD-DFT/MM–calculated emission for electronic transition between S_1_ and S_0_ for the model GB_Av_-closed-R337L (resulting from the R337L mutation in GB_Av_-closed before MD). The TD-DFT/MM calculations were performed with the 6-311G(2d,p) basis set and the B3LYP functional on a structure optimized at the same level of theory.

Table S5 TD-DFT/MM emission for electronic transition between S_1_ and S_0_ for the model GB_Av_-closed-R337L/I351L (resulting from double R337L and I351L mutations in GB_Av_-closed before MD). The TD-DFT/MM calculations were performed with the 6-311G(2d,p) basis set and the B3LYP functional on a structure optimized at the same level of theory.

**Figure S8.**
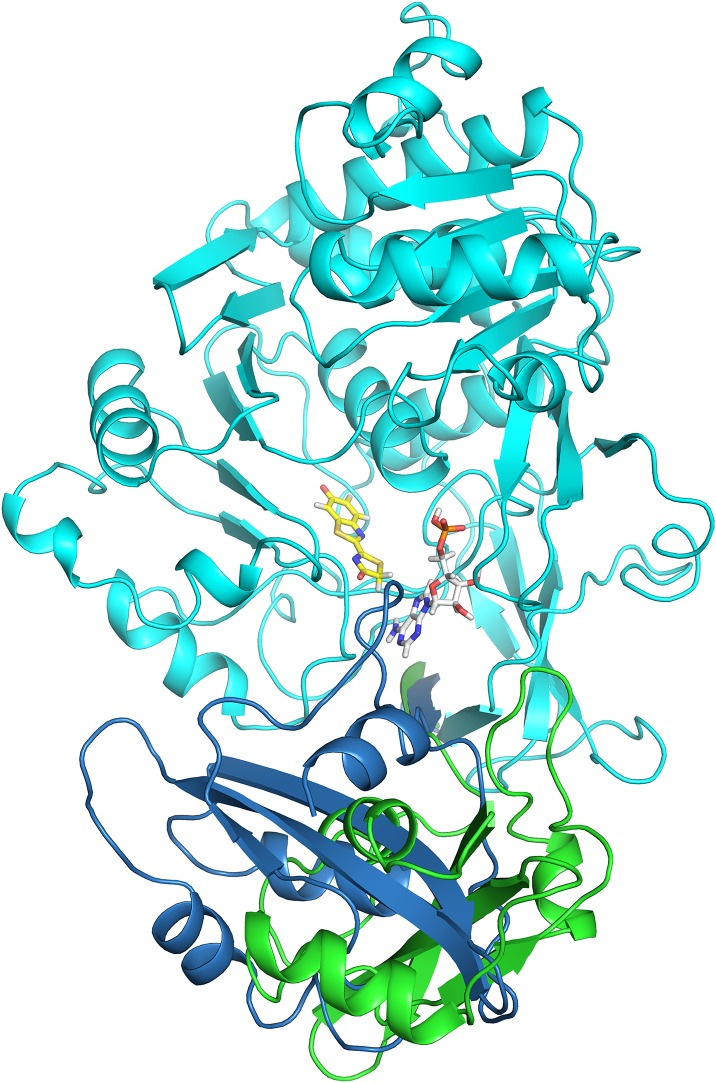
Superposition of the N-terminal domains of the “open” (GB_Av_-open) and the modeled “closed” (GB_Av_-closed) states of GB_Av_. The C-terminal domains of GB_Av_-closed (blue) and GB_Av_-open (green) are displaced with respect to the N-terminal domain. In the classical MD simulation performed with the GB_Av_-closed model, the oxyluciferin adopts a different position in the active site of GB_Av_-closed relative to simulation with green-emitting luciferases (Fig S9). The reorganization of the electrostatic potential exerted by the surrounding residues of GB_Av_ leads to a blue shift of the emission. As a result of its new position, the oxyluciferin is distant from S250 of GB_Av_, the only residue of the active site that is substituted in the green luciferases (Fig S9). A hydrogen bond between S250 and oxyluciferin in GB_Av_-closed was not observed. Consequently, the presence of S250 alone cannot explain the observed blue shift of the emission of GB_Av_.

**Figure S9.**
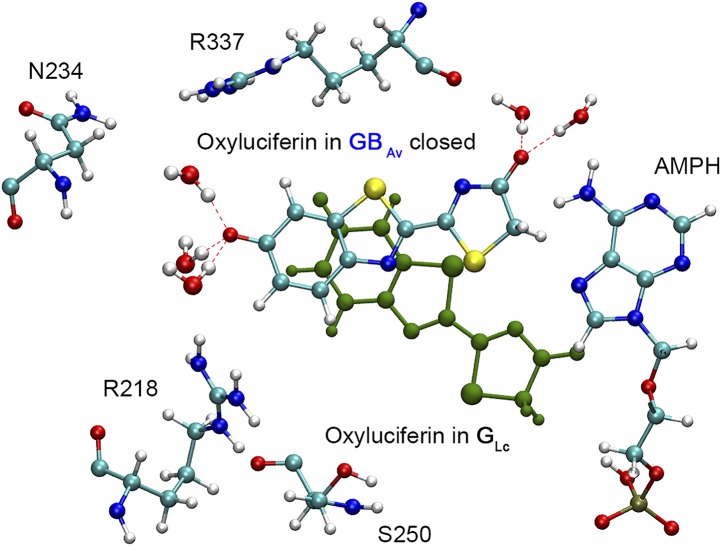
Ball-and-stick representation of the superposed structures of the GB_Av_-closed model with oxyluciferin and the crystal structure of G_Lc_ luciferase (green) with oxyluciferin that shows the oxyluciferin-binding pocket. The structures are superimposed in the N-terminal domains of GB_Av_-closed from snapshot 2 and the crystal structure of G_Lc_ luciferase. The GB_Av_-closed model is represented as color-coded atoms, whereas the oxyluciferin from G_Lc_ is in green. The AMPH is represented in color-coded atoms.

**Figure S10.**
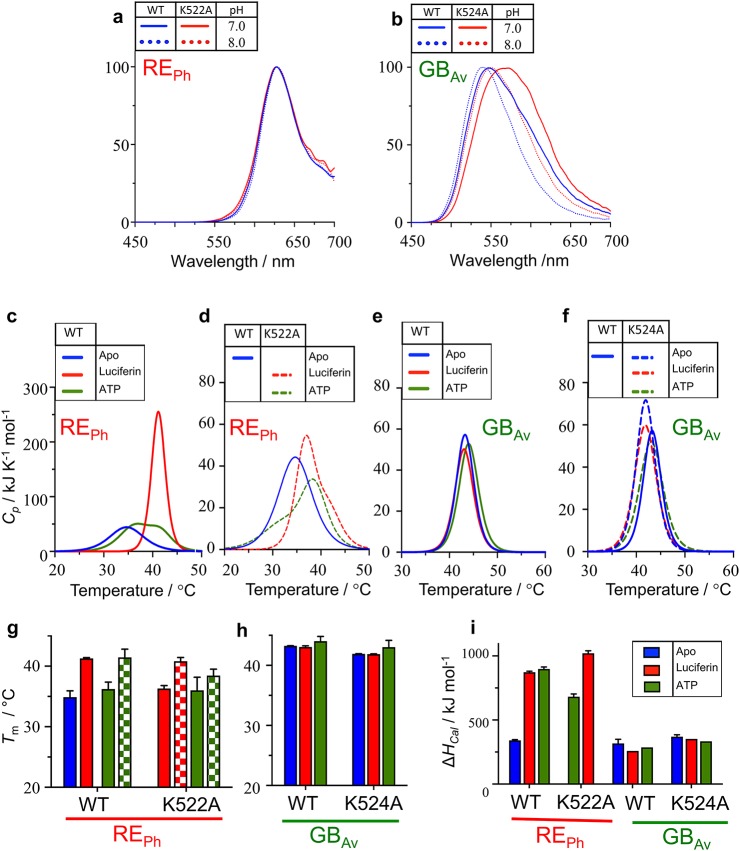
Normalized bioluminescence emission spectra and thermal analysis (DSC) of RE_Ph_ and GB_Av_ luciferases. **(A)** The normalized emission spectrum of WT RE_Ph_ is independent of pH, with λ_max_ = 623 nm. The single mutant K522A did not shift the WT emission. **(B)** The emission of GB_Av_ is pH-dependent, and introduction of the K524A mutation red-shifts the WT emission by ≈18 nm, from 550 to 573 nm at pH 7.0, and from 538 to 555 nm at pH 8. **(C–F)** DSC thermograms of RE_Ph_ and GB_Av_ luciferases, WT (solid line), and mutants (broken line). The DSC measurements were acquired in the absence (blue) or presence of firefly 5 mM luciferin (red) or 5 mM ATP (green). The DSC scans were corrected for buffer baseline and the data were fitted to non–two-state transitions that show cooperative endothermic unfolding. RE_Ph_ is thermodynamically less stable than GB_Av_. However, addition of substrates enhances the stability of RE_Ph_ but does not alter that of GB_Av_. **(G–H)** Bar plot of the first (solid bars) and second (checkered bars) melting points calculated from the temperatures at the middle of the first and second transition of the DSC thermograms, respectively. **(I)** Bar plot of Δ*H*_cal_ determined from the area under the peaks in the absence (blue) or presence of firefly luciferin (red) or ATP (green). The data are shown as mean ± SD, with *n* = 3.

Supplementary Note 4 *Computational study of WT GB*_*Av*_.
To understand the natural blue shift of the light observed for GB_Av_ relative to common green-yellow emitting luciferases, computational analyses were performed with one of the monomers extracted from the crystal structure of GB_Av_. The model was generated by following the steps outlined in the method section.
As noted in the main text, GB_Av_ has its C-terminal domain in an open conformation. Two models were analyzed: one model with an open C-terminal domain (“GB_Av_-open”) and one model was constructed with its C-terminal domain closed (“GB_Av_-closed”; Supplementary Fig. 9). The closure of the C-terminal domain was simulated by using an umbrella sampling classical molecular dynamics (MD), as described in the method section. Random snapshots were extracted from 10 ns classical MD simulations on both models, and the value of the fluorescence emission maximum was calculated by time-dependent density functional theory/molecular mechanics (TD-DFT/MM).
The emission calculated from snapshots in GB_Av_-open shows a red shift relative to the experimental value (Supplementary Table 2). The most pronounced difference is 15 nm (0.06 eV). These results — accounting for the level of theory employed (TD-DFT/MM) and the intrinsic error of the method — show good agreement with the experimental emission recorded for GB_Av_. However, the calculated values are also close to the experimental emissions of other green-emitting firefly luciferases, which include the luciferases of *Luciola cruciata* at 560 nm (PDB code: 2D1R) and *Photinus pyralis* at 558 nm (PDB code: 4G36). Therefore, the crystal structure of GB_Av_ in the open conformation did not show the expected blue-shifted emission of GB_Av_.
The umbrella sampling classical MD simulations used to close the C-terminal domain and to obtain GB_Av_-closed has a flat free-energy profile, which reveals the absence of an energy barrier. The TD-DFT/MM-calculated emission of the resulting GB_Av_-closed model is slightly blue-shifted relative to the experimental value and further from the green-emitting firefly luciferase wavelengths. The largest difference is 13 nm (0.06 eV) and therefore the closed model better reproduced the experimental blue shift observed with GB_Av_ luciferase.
During the classical MD simulation performed on GB_Av_-closed, the oxyluciferin adopts a different position in the cavity than the one observed in simulations with models of green-emitting firefly luciferases (Supplementary Fig. 10). The reorganization of the electrostatic potential exerted by the residues that surround the active site in GB_Av_ leads to a blue shift in emission. As a result of its new position, oxyluciferin is far from S250 in GB_Av_, the only residue of the active site that differs from the green-emitting luciferases (Supplementary Fig. 8). No hydrogen bond was observed between S250 and the oxyluciferin in GB_Av_-closed. Consequently, the sole presence of S250 alone cannot explain the observed blue shift in GB_Av_ luciferase.
To further understand the effect of residue S250 on the emission of GB_Av_, the corresponding *in silico* mutation F251S was included in the model G_Lc_ derived from the luciferase of *Luciola cruciata* (PDB code: 2D1R). The calculated emission of the F251S mutant of the G_Lc_ model shows a surprisingly small red shift, probably as a result of formation of a H-bond network between S251, a water molecule, and oxyluciferin. The calculated value of the emission is 566 nm (2.19 eV). The result indicates that the substitution of phenylalanine in G_Lc_ to serine in GB_Av_ is not responsible for the experimentally observed blue shift.
*Computational study of mutations in GB*_*Av*_ *and comparison with RE*_*Ph*_.
To identify the reasons behind the red-shifted emission of RE_Ph_ relative to GB_Av_, selected amino acid residues in GB_Av_ were mutated to those found in RE_Ph_. Three residues were found to be important for the difference between these two luciferases. Two mutations are on residues located in the active site, R337 and I351 in GB_Av_ that correspond to L334 and L348 in RE_Ph,_ respectively. To model the effect of these substitutions, we took snapshot 1 of the GB_Av_-closed model and manually replaced the residues to perform a 10 ns classical MD (three different simulations, with R337L, I351L, and double mutant R337L/I351L). Several snapshots were extracted from the MD calculation and the respective fluorescence electronic transitions were calculated.The single mutation, I351L, has nearly no effect on the emission of GB_Av_-closed, similar to the experimental results (Fig. 3 and Supplementary Table 3). As predicted and in accordance with the similar properties of these residues, the mutant I351L did not alter the emitted color. For the other single mutant, R337L, a red shift in emission was observed after a simulation time of 6.8 ns, with a red shift between 25 and 45 nm relative to GB_Av_-closed (Supplementary Table 4). The geometry of the active site was conserved, and no major changes in the oxyluciferin binding pocket were observed. However, during the MD simulation, R218 moved and formed a hydrogen bond with oxyluciferin (Supplementary Fig. 10). An analysis of the emission of the calculated value for single mutant R337L and the experimental values of the emission of single mutant R337L and double mutant R337L/I351L showed good agreement (snapshot 4 gives a value of 577 nm that is less than 5 nm different from the experimental value of 580 nm; Supplementary Table 4). As expected, the substitution of a charged residue close to oxyluciferin with an aliphatic residue has a strong effect on the emission.
The double mutant, R337L/I351L, shifted the emission to the red after a MD simulation time of 7 ns (Supplementary Table 5). A large red-shift (>50 nm) was observed for this double mutant relative to GB_Av_-closed. This shift is not related to the geometry of the active site, which was conserved during the MD simulation without major changes in the position of oxyluciferin. However, displacement of R218 to form hydrogen bond with oxyluciferin was observed again. The calculated emissions are in a good agreement with the experimental results of double mutant R337L/I351L (See Table 5 in which experimental value = 580 nm, and calculated value = 595 nm). In the double mutation, mutation R337L appears to be more important relative to I351L to explain the red shift.
Finally, the natural insertion R353 in loop^348‒363^ of RE_Ph_ was studied in GB_Av_-closed_._ The presence of R353 appears as the most viable reason for the conformational changes observed for loop^348‒363^, including its high *B* factor. To model this motion, arginine was inserted at position 356 in snapshot 1 of GB_Av_-closed. A longer MD simulation of 30 ns was performed to extract several snapshots. A small red shift of ≈ 15 nm after 20 ns was observed (Supplementary Table 6). Even after a simulation time of 30 ns, the insertion of R356 did not change the conformation of the loop, in contrast with its position in the crystal structure of RE_Ph_. This result may be due to multiple amino acid substitutions that are responsible for the motion of loop^351‒364^, which includes the substitution of E354 in GB_Av_ with N351 in RE_Ph_ (Fig. 2e and 2f).

### Loop^523–530^ only contributes to the green emission of GB_Av_

A second highly conserved loop in beetle luciferases at the end of the C-terminal domain, loop^523–530^ in GB_Av_ (loop^521–528^ in RE_Ph_), is also relevant to the aperture of the enzyme (Figs S4 and S5). Despite the low resolution of the RE_Ph_ structure, the high-quality electron density map allowed to model this loop region in the *P*3_1_21 crystal form (Fig S1D), where rotation and displacement of loop^523–530^ by ∼10 Å closes the active site and facilitates interactions of key residues with the substrates. In the closed state of G_LC_, K526 interacts with T292 and D468 of the N- and C-terminal domains, respectively (Fig S4E). Upon closure of the active site, T527 and K529 of GB_Av_ are shifted to interact with the substrate in accordance with their catalytic roles in the bioluminescence reaction (Branchini et al, 2000, 2004, 2005). To assess the role of the conformation of the C-terminal domain on the emission from the excited state of the product, a complementary umbrella classical molecular dynamics simulation was performed on GB_Av_ in which K524 of the C-terminal domain was constrained to interact with the backbone of G311 of the N-terminal domain to close the active site in GB_Av_ (Figs S5, S8, and S9 and Supplementary Note 4). The results revealed that rotation of the C-terminal domain between the open and closed states is not related to energetic barrier. Moreover, the bioluminescence emission calculated for the closed state (530 ± 10 nm) is in strong agreement with the experimental value of 538 nm (Table S6). Thus, the presence of loop^523–530^ inside the active site of the luciferase is required for the natural bioluminescence color of WT GB_Av_.

Table S6 TD-DFT/MM emissions corresponding to the electronic transition between S_1_ and S_0_ for GB_Av_-open and GB_Av_-closed. The TD-DFT/MM calculations were performed with the 6-311G(2d,p) basis set and the B3LYP functional on a structure optimized at the same level of theory.

We were able to discern with sufficient accuracy the structure of loop^523–530^ in both independent molecules in the crystal of GB_Av_, in which the open conformation is stabilized by interactions between T527 and K529 of loop^523–530^, and D422 and D436 of the N-terminal domain (Fig S4C). The structural integrity of loop^523–530^ is maintained by interaction between K524 and the backbone of G528. The mutant K524A in loop^523–530^ of GB_Av_ exhibited a red shift of 12 nm with minimum decrease in its thermodynamic stability (Figs 3C and D, S10B, E, F, and G–I, and Supplementary Note 3). The emission of the same mutant (K522A) of RE_Ph_ remained unaltered, even though its thermodynamic stability decreased (Figs 3C, S10A, C, D, and G–I). With the exception of T525A in RE_Ph_, which retained ≈ 40% of its emission intensity, other mutants in loop^523–530^, which include T527A and K529A of GB_Av_, and T525A and K527A of RE_Ph_, suppressed the WT enzyme emission completely.

The relevance of the C-terminal domain in the color-tuning mechanism was further explored by introducing mutation K524A, which is distant from the active site in both the open and closed conformations (Fig 3E). The slightly decreased thermodynamic stability of this mutant indicates minor destabilization of the interactions of K524 with T290 and D466 (as seen in the structure of GB_Av_), which are at the interface between the N- and C-terminal domains in the closed state (Fig S4E and Supplementary Note 3). This result is similar to the red shift in emission of other mutations that have been introduced previously in the C-terminal domain and are located on the same interface (Fig 3). This interface between the N- and C-terminal domains includes polar and hydrophobic interactions that, when disrupted, are known to shift the color to red by ≈10–15 nm without affecting the general fold of the enzyme (Modestova & Ugarova, 2016).

## Discussion

The structural determination of the only naturally red-emitting RE_Ph_ has, for the first time, revealed an oligomeric structure for a beetle luciferase, whereas others are all exclusively monomeric. Although oligomerization is possible in solution, it is not critical for the emission of red light; instead, the low emission energy is inherent to the structure of the monomer and the microenvironment of the active site. Although co-crystals with either substrate could not be achieved, the structures of RE_Ph_ with red emission and GB_Av_ with blue-shifted green emission provide insight into the effects that conformational changes and active site microenvironment have on the color of the light emitted by luciferases. Furthermore, two conserved segments, loop^351–364^ in GB_Av_ and loop^348–361^ in RE_Ph_, were identified by mutagenesis to have a profound effect on the emission of both enzymes. The strong interactions of R337 with loop^351–364^ in GB_Av_ are important for its green emission. Similarly, mutations that altered the interactions with loop^348–361^ blue-shift the emission of RE_Ph_ from 623 nm, for the WT, to 605 nm in the L334R mutant. The second conserved segment, loop^523–530^ in GB_Av_ or loop^521–528^ in RE_Ph_, was found to be important for the emission of GB_Av_ only, and substitutions in loop^521–528^ did not alter the red emission of RE_Ph_. These results provide direct insight into the molecular origin of the diverse colors emitted by different beetle luciferases and are the key to solving one of the most difficult conundrums in bioluminescence research.

## Materials and Methods

### Materials

Unless mentioned otherwise, the chemicals were from Sigma-Aldrich. The synthesis of full-length genes and oligonucleotide DNA primers, DNA sequencing, and, in few cases, site-directed mutagenesis were carried out by GenScript USA Inc. Restriction enzymes were purchased from New England Biolabs Inc., and KOD DNA polymerase and dNTP mix were from EMD Millipore.

### Cloning, expression, and purification

The genes of the natural red-emitting luciferase from *P. hirtus* (RE_Ph_; railroad worm) and the green-emitting luciferase with blue-shifted emission (GB_Av_) from *A. vivianii* were synthesized by GenScript (GenScript USA Inc.). The DNA sequences, based on the previously reported proteins sequences (Viviani et al, 1999, 2011), were optimized for *Escherichia coli* expression and designed to be subcloned into a pET26b-derived bacterial expression vector that contains an N-terminal domain SUMO fusion protein tag (Champion pET SUMO system, Thermo Fisher Scientific) by using NheI and XhoI as restriction sites. Further subcloning was performed into selected pET28-derived systems by using NheI–HindIII restriction sites (RE_Ph_) and BamHI–HindIII (GB_Av_). The best overexpression results were obtained for GB_Av_ cloned into the pET28a+ system (Novagen), whereas the RE_Ph_ expression was improved by using the pET28 MHL system (provided by the Structural Genomics Consortium). Most of the point mutations were introduced by site-directed mutagenesis (Edelheit et al, 2009), whereas others were ordered from GenScript (all point mutations used to destabilize the octamer).

The luciferase constructs were introduced by transformation into *E. coli* BL21-CodonPlus-RIL (Stratagene). A single colony was used to inoculate Luria broth that contains kanamycin (100 mg/l) and chloramphenicol (50 mg/l) and grown overnight at 37°C with vigorous shaking (preinocules). The inoculated cultures (usually 4–6 liters for each construct) were grown at 37°C until the *A*_600_ reached 0.2. At this point, the temperature was lowered to 28°C until an *A*_600_ of 0.3 was achieved, and the luciferases expression was then induced overnight (at 15°C) by adding IPTG (0.2 mM). The cells were harvested by centrifugation at 12,000 *g* for 10 min in an Avanti J26-XPI centrifuge (Beckman Coulter Inc.), then resuspended in lysis buffer (Tris [100 mM], pH 7.8, NaCl [500 mM], glycerol [10%], imidazole [5 mM], βME [3 mM], and protease inhibitor cocktail from Sigma-Aldrich: P8849), lysed by sonication on ice, and centrifuged again at 40,000 *g* rpm for 30 min at 4°C. The supernatants were loaded at a rate of 1 ml/min onto a ProBond Nickel-Chelating Resin (Life Technologies) previously equilibrated with binding buffer (Tris [100 mM], pH 7.8, NaCl [500 mM], glycerol [10%], imidazole [5 mM], and βME [3 mM]) at 4°C. The columns were washed with 10 column volumes (cv) of binding buffer, followed by 15 cvs of washing buffer (Tris [100 mM], pH 7.8, NaCl [250 mM], glycerol [10%], βME [3 mM], and imidazole [50 mM]). Recombinant luciferases were eluted by using elution buffer (Tris [100 mM], pH 7.8, NaCl [500 mM], glycerol [10%], βME [3 mM], and imidazole [300 mM]). Finally, the proteins were loaded onto a HiLoad Superdex S200 size-exclusion filtration column (GE Healthcare), attached to an AKTΔpurifier core system (GE Healthcare), and pre-equilibrated with filtration buffer (Hepes [20 mM], pH 7.8, NaCl [500 mM], glycerol [10%], and TCEP [1 mM]). The final protein peaks were collected and concentrated to ≈5–10 mg/ml (measured by absorbance) and their purity was analyzed by using SDS–PAGE.

### Crystallization

A high-throughput approach was used to explore initial crystallization conditions for RE_Ph_ (7.2 mg/ml) and GB_Av_ (5.6 mg/ml) luciferases that were previously purified. Commercially available conditions from screens PACT suite, JCSG (Qiagen), INDEX, and Crystal Screen 1 and 2 (Hampton Research), were tested by using a vapor diffusion method on sitting drops. A total of 75 μl of each condition was dispensed into a 96-well SD2 Molecular Dimensions (MD2) plate by a Bravo robot (Agilent Technologies). A volume of 250 nl of protein was mixed (1:1) with each condition by using a Honeybee X8 robot (Isogen Life Science). The plates were sealed, incubated at 18°C, and checked regularly under the microscope. Along with the free enzymes, several complexes that include the enzyme and ATP (in presence of MgCl_2_), enzyme and luciferin, and enzyme and ATP and luciferin (supplemented with MgCl_2_) were tested at different ligand concentrations (0.1–5.0 mM).

The hits obtained for both enzymes were systematically explored to improve the initial crystals by making 2D variations of the pH and all the components (precipitant and additives) in 96-well plates, and manually in MRC Maxi 48-Well Crystallization Plate (Swissci). The improved conditions were scaled to 1.5 μl drops (protein/precipitant = 2:1) and bigger single crystals were obtained for both GB_Av_ and RE_Ph_ (free enzymes). The final crystallization conditions were sodium citrate (1.6 M), pH 6.5, supplemented with MgCl_2_ (200 mM) for GB_Av_, and polyethylene glycol 3350 (19–21%), bis–tris propane (100 mM), pH 6.0, supplemented with ammonium sulfate (200 mM) for RE_Ph_.

### X-ray data collection and structure determination

The crystals of both proteins were fast-frozen in liquid nitrogen. Only the crystals of RE_Ph_ were soaked into a cryoprotectant solution (crystallization solution supplemented with 15–20% glycerol) as the final crystallization condition for GB_Av_ crystals (sodium citrate [1.6 M], pH 6.5) worked as a cryoprotectant solution. The diffraction data were collected by using synchrotron radiation at the Swiss Light Source (beamline X06DA) with a Pilatus 2M detector (Dectris) and at the MX2 beamline from the Australian Synchrotron with a Quantum 315r Detector (ADSC). Routinely, 360^o^ were collected for each crystal with an oscillation range between 0.1 and 0.5° and exposure time of 0.1–1 s per image, depending on the quality of crystals. Several datasets were collected for GB_Av_ (free enzyme) up to 1.9 Å (Fig 1B), others for RE_Ph_ (free enzyme) up to 3.1 Å at X06DA and up to 3.6 Å at MX2 beamline (Australian Synchrotron). Other datasets were collected for crystals of GB_Av_ soaked with ATP (up to 2.2 Å). The crystals of RE_Ph_ are in the triclinic space group *P*1 (parameters: *a* = 105.70 Å; *b* = 121.17 Å; *c* = 129.44 Å; α = 61.86°; β = 68.35°; γ = 74.17°) and trigonal *P*3_1_21 (parameters: *a* = *b* = 119.100 Å; *c* = 351.402 Å; α = β = 90.00°; γ = 120.00°), whereas crystals of GB_Av_ are in the orthorhombic space group *P*2_1_2_1_2_1_ (parameters: *a* = 94.28 Å; *b* = 110.53 Å; *c* = 122.07 Å; α = β = γ = 90°).

For the *P*1 crystal form of RE_Ph_, two isomorphous datasets were merged to obtain a good dataset at 3.05 Å (Table S1), which was used to solve the structure. The datasets were indexed and integrated by using XDS (Kabsch, 2010) and iMosflm (Battye et al, 2011) and scaled with Scala (Evans, 2006) from the CCP4 program suite (Winn et al, 2011). The protein structures were phased by molecular replacement using the program Molrep (Vagin & Teplyakov, 1997) within the molecular replacement protocol of Auto-Rickshaw (Panjikar et al, 2005, 2009). The N-terminal domain of the Japanese firefly structure (PDB code: 2D1R) was used as a template. Refinement of the structures was carried out with the PHENIX program (Afonine et al, 2012). As a result of differences in the conformation of the C-terminal domains, this part of the structure was manually built in both molecules of GB_Av_ found in the asymmetric unit, and later in the only complete monomer of RE_Ph_ using the program Coot (Emsley et al, 2010). An iterative process of manual building and refinement with the PHENIX program (Afonine et al, 2012) was applied to both structures. Data processing and refinement statistics are summarized in Table S1. The final models presented good stereochemistry parameters and *R* and *R*_free_ values. Also, both RE_Ph_ crystal forms showed good-quality electron density maps for their low resolution (Fig S1A and B). The figures of the structures were generated with PyMol (DeLano, 2002). Structure factors and atomic coordinates are deposited in the Protein Data Bank (PDB accession codes: 6AAA, 6ABH, and 6AC3).

### Differential scanning calorimetry (DSC)

Calorimetric analyses for the WT proteins and mutants were performed by using a Nano-DSC (TA Instruments). The samples and buffers were degassed under vacuum for 15 min with stirring at 10°C. The concentrations of all the proteins were adjusted to 0.5 mg/ml. A total volume of 300 μl of each degassed sample in Hepes (20 mM; pH 7.8) and MgCl_2_ (10 mM) was loaded into the sample cell. For measurements with ligands, ATP and firefly luciferin (Gold Biotechnology) were added to both, the sample and reference cell in the same concentration (5 μM). The samples were heated at a scan rate of 1°C/min from 10 to 80°C at 3 atm. Before loading the samples, background scans were obtained by loading degassed buffer (with or without substrates) in both the reference and samples cells and heated at the same rate. The enthalpies of the transitions (Δ*H*_cal_) were estimated by calculating the area under the thermal transition after subtracting the blank and fitting a baseline by using Nano Analyzer software provided by the manufacturer.

### Computational analysis

The closed state of GB_Av_ was modeled with classical MD and quantum mechanics (QM)/MM calculations. To integrate the oxyluciferin in its keto form and protonated adenosine 5′-monophosphate nucleotide (AMPH) molecules in the active site of GB_Av_, manual docking was performed based on the position of these two molecules in the structures of the North American firefly, *P. pyralis* (PDB code: 4G37), and the Japanese firefly, *L. cruciata* (PDB code: 2D1R). All calculations were performed on one of the molecules (chain B) in the GB_Av_ structure.

The residues were protonated by using Leap from Amber14 suite of the program (Case et al, 2017). The contentious cases, especially for histidines, were resolved by computing their p*K*_a_ with the H++ program (DeLano, 2002) to have a neutral charge for the system. Depending on the model considered, we selected to double-protonate (i.e., one hydrogen on each nitrogen of the side chain, which resulted in a positively charged residue) the following histidines for luciferases in complex with oxyluciferin and AMPH:

(1) GB_Av_-open with, GB_Av_-closed, and GB_Av_-closed-I347L: the doubly protonated histidines are 5, 23, 42, 72, 305, 404, and 426.
(2) GB_Av_-closed-R337L: the doubly protonated histidines are 5, 23, 42, 72, 305, 404, 426, and 456.
(3) GB_Av_-closed-insert-R356: the doubly protonated histidines are 5, 23, 42, 72, 405, and 427.

AMPH was included with a single negative charge, and oxyluciferin was modeled in its phenolate-keto form, with a single negative charge.

Classical dynamics simulations were performed with Amber14 to obtain several snapshots for further QM/MM optimization. The model was solvated with TIP3P water molecules within a cubic box by ensuring a solvent shell of at least 15 Å around the solute. The resulting system contained ≈28,000 water molecules and 90,000 atoms in total. The AMBER99ff was used to model the residues of the protein. The AMPH and the emitter (oxyluciferin) were described by using parameters developed by the Navizet group (Navizet et al, 2010, 2011; Chen et al, 2011; Anandakrishnan et al, 2012). The parameters were not fully optimized for the excited state, so the oxyluciferin structure was first obtained by QM/MM optimization of the first singlet excited state and frozen in its excited state conformation during the whole dynamics simulation. The system was heated from 100 to 300 K in 20 ps. Then, under isothermal-isobaric ensemble (NPT) conditions with *T* = 300 K and *P* = 1 atm, a 10-ns dynamic with periodic boundary conditions was realized with a 2-fs time step. During these simulations, the pressure and temperature were maintained by using the Langevin algorithm with a coupling constant of 5 ps. SHAKE constraints were applied to all bonds that involved hydrogen atoms (Ryckaert et al, 1977). Random snapshots were extracted along the MD and used to compute the QM/MM emission. These snapshots correspond to low-energy points of the classical MD.

The folding of the C-terminal domain was performed by using umbrella sampling MDs (Kästner, 2011). In detail, the umbrella sampling was realized between the two α-carbons of residues 311 and 524. The distance is 21 Å at the beginning and ends at 7 Å, with a step of 1 Å. For each step, an equilibration of 200 ps followed by a 750-ps production was realized. The lowest energy conformation was also collected for each step. This yields a set of structures along the path that decreases the distance between the two α-carbons of residues 311 and 524. The bias introduced by the umbrella potential was removed by using the weighted histogram analysis method (Kumar et al, 1995; Roux, 1995), to generate a free-energy profile along the approach path.

The QM/MM calculations were performed by using a QM/MM coupling scheme between Gaussian (Frisch et al, 2016) and Tinker (Tinker, 2005) (Gaussian 09d/Tinker). The electrostatic potential fitted method (Ferré & Ángyán, 2002) was used to compute the interaction between the Mulliken charges of the QM subsystem and the external electrostatic potential of the MM subsystem within 9 Å from the QM part. The microiterations technique (Melaccio et al, 2011) was used to converge the MM subsystem geometry for every QM minimization step. The emitter was selected as the QM subsystem, whereas the rest of the system was assigned to the MM subsystem. The QM/MM optimization of the first singlet excited state (S_1_) was performed first, followed by calculation of the vertical difference of energies between S_1_ and the ground state (S_0_), which corresponds to the fluorescence emission. In fireflies, the fluorescence transition (induced by photoexcitation) is the same as the bioluminescence transition (obtained as a result of a bioluminescence reaction) (Navizet et al, 2013); therefore, the calculated emission value can be equated to the experimental emission energy.

The levels of theory (the treatment of the electron correlation and the basis set) used in the QM of the QM/MM calculations were chosen as follows: the time-dependent density functional theory (TD-DFT) calculations were carried out by using the B3LYP functional with the 6-311G(2d,p) basis set. The selected basis set was 6-311G(2d,p); we did not use basis set with diffuse functions because they can interact with the MM system with electrostatic embedding (electrostatic potential fitted method). As detailed in reference (Berraud-Pache & Navizet, 2016), these conditions are optimal for this type of calculation.

## Supplementary Information

Supplementary Information is available at https://doi.org/10.26508/lsa.201800072.

## Supplementary Material

Reviewer comments
