# Supplementary material for: Beetle luciferases with naturally red- and blue-shifted emission
Source: Life Sci Alliance. 2018 Aug 16;1(4):e201800072. doi: 10.26508/lsa.201800072 (PMC6238593; doi:10.26508/lsa.201800072)
Supplement: Supplementary file 1 [file LSA-2018-00072_TableS1.docx]

**Supplementary Table 1**. Data collection and refinement statistics.

|  | **Structure** | | | |
| --- | --- | --- | --- | --- |
|  | GB_Av_ | | RE_Ph_  (*P*1) merged | RE_Ph_  (*P*3_1_21) |
| **Data collection and unit cell dimensions*^a^*** | | | | |
| Wavelength (Å) | 1.0 | 1.0 | | 1.0 |
| Synchrotron | SLS | SLS | | Australian synchrotron |
| Space group | *P*2_1_2_1_2_1_ | *P*1 | | *P*3_1_21 |
| *a*, *b*, *c* (Å) | 94.28, 110.53, 122.07 | 105.74, 121.26, 129.55 | | 119.10, 119.10, 351.40 |
| *α*, *β*, *γ* (°) | 90, 90, 90 | 61.85, 68.30, 74.16 | | 90.00, 90.00, 120.00 |
| Resolution (Å) | 1.9 | 3.05 | | 3.6 |
| No. of reflections | 1,027,690 | 517,157 | | 186,010 |
| No. of unique reflections | 100,333 | 98,610 | | 33,877 |
| *R*_merge_ | 0.108 (1.432) | 0.111 (1.046) | | 0.211 (0.936) |
| R_pim_ | 0.035 (0.488) | 0.053 (0.497) | | 0.109 (0.488) |
| CC_1/2_ | 0.999 (0.706) | 0.998 (0.769) | | 0.985 (0.504) |
| Mean I/σ (I) | 17 (1.8) | 14.5 (2.0) | | 6.5 (2.0) |
| Completeness (%) | 100 (100) | 98.9 (98.9) | | 99.1 (99.1) |
| Redundancy | 10.2 (9.4) | 5.2 (5.3) | | 5.5 (5.4) |
| **Refinement** | | | | |
| Resolution (Å) | 28.08‒1.90 (2.00‒1.90) | 48.76‒3.05 (3.16‒3.05) | | 19.85‒3.6 (3.78‒3.6) |
| *R*_work_/*R*_free_ (%) | 19.2/21.9 | 24.5/28.5 | | 20.6/28.1 |
| No. of atoms |  |  | |  |
| Protein | 8,377 | 26,183 | | 13,930 |
| Ligand (Glycerol) | 1 |  | |  |
| Water | 783 |  | |  |
| Protein residues | 1,075 | 3,343 | | 1,834 |
| *B* factors (Å^2^) |  |  | |  |
| Protein | 43.60 | 93.30 | | 63.04 |
| Ligand (Glycerol) | 75.20 |  | |  |
| Water | 44.50 |  | |  |
| R.m.s deviations |  |  | |  |
| Bond lengths (Å) | 0.003 | 0.012 | | 0.012 |
| Bond angles (°) | 0.758 | 1.62 | | 1.64 |
| Ramachandran favored (%) | 97 | 87 | | 87 |
| Ramachandran outliers (%) | 0 | 4.3 | | 4.5 |
| PDB accession code | 6AAA | 6ABH | | 6AC3 |

*^a^*Values within parenthesis are for the highest resolution shell.
