# Supplementary material for: Beetle luciferases with naturally red- and blue-shifted emission
Source: Life Sci Alliance. 2018 Aug 16;1(4):e201800072. doi: 10.26508/lsa.201800072 (PMC6238593; doi:10.26508/lsa.201800072)
Supplement: Supplementary file 3 [file LSA-2018-00072_TableS3.docx]

**Supplementary Table 3**. TD-DFT/MM emission for electronic transition between S_1_ and S_0_ for the model GB_Av_-closed-I351L (resulting from the I351L mutation in GB_Av_-closed before MD). The TD-DFT/MM calculations were performed with the 6-311G(2d,p) basis set with the B3LYP functional on a structure optimized at the same level of theory.

| Model*^a^* | TD-DFT/MM  in eV*^b^* |
| --- | --- |
| Snapshot (1) | 2.35 (528) |
| Snapshot (2) | 2.40 (517) |
| Snapshot (3) | 2.33 (531) |
| GB_Av_-closed snapshot (1) of Supplementary Table 2 | 2.32 (535) |
| GB_Av_ mutant I351L (experimental value) | 2.30 (538) |

*^a^*The values in parentheses represent the number of the snapshot extracted from the corresponding MD simulation.

*^b^*The associated wavelengths in nm are given in parentheses.
