# Supplementary material for: Beetle luciferases with naturally red- and blue-shifted emission
Source: Life Sci Alliance. 2018 Aug 16;1(4):e201800072. doi: 10.26508/lsa.201800072 (PMC6238593; doi:10.26508/lsa.201800072)
Supplement: Supplementary file 5 [file LSA-2018-00072_TableS5.docx]

**Supplementary Table 5**. TD-DFT/MM emission for electronic transition between S_1_ and S_0_ for the model GB_Av_-closed-R337L/I351L (resulting from double R337L and I351L mutations in GB_Av_-closed before MD). The TD-DFT/MM calculations were performed with the 6-311G(2d,p) basis set and the B3LYP functional on a structure optimized at the same level of theory.

| Model*^a^* | TD-DFT/MM  in eV*^b^* |
| --- | --- |
| Snapshot (1) [simulation times = 7 ns] | 2.08 (595) |
| Snapshot (2) [simulation times = 9.5 ns] | 2.10 (590) |
| GB_Av_-closed snapshot (1) of Supplementary Table 2 | 2.32 (535) |
| GB_Av_ double mutant R337L/I351 (experimental value) | 2.13 (580) |

*^a^*The values in parentheses represent the number of the snapshot extracted from the corresponding MD simulation.

*^b^*The associated wavelengths in nm are given in parentheses.
