# Supplementary material for: Beetle luciferases with naturally red- and blue-shifted emission
Source: Life Sci Alliance. 2018 Aug 16;1(4):e201800072. doi: 10.26508/lsa.201800072 (PMC6238593; doi:10.26508/lsa.201800072)
Supplement: Supplementary file 6 [file LSA-2018-00072_TableS6.docx]

**Supplementary Table 6**. TD-DFT/MM emissions corresponding to the electronic transition between S_1_ and S_0_ for GB_Av_-open and GB_Av_-closed. The TD-DFT/MM calculations were performed with the 6-311G(2d,p) basis set and the B3LYP functional on a structure optimized at the same level of theory.

| Model (numbering of the snapshot)*^a^* | TD-DFT/MM emission transition in eV*^b^* | Experimental value for GB_Av_ in eV*^b^* |
| --- | --- | --- |
| GB_Av_-open (1) | 2.24 (552) | 2.3 (538) |
| GB_Av_-open (2) | 2.25 (551) |  |
| GB_Av_-open (3) | 2.24 (553) |  |
| GB_Av_-open (4) | 2.29 (541) |  |
| GB_Av_-closed (1) | 2.32 (535) |  |
| GB_Av_-closed (2) | 2.33 (532) |  |
| GB_Av_-closed (3) | 2.36 (525) |  |
| GB_Av_-closed (4) | 2.35 (527) |  |

*^a^*The values in parentheses represent the number of the snapshot extracted from the corresponding MD simulation.

*^b^*The associated wavelengths in nm are given in parentheses.
